# COVID-19 vaccine hesitancy among first-generation immigrants living in Sweden

**DOI:** 10.1093/eurpub/ckad073

**Published:** 2023-05-25

**Authors:** Signe Svallfors, Elin C Larsson, Bi Puranen, Anna Mia Ekström

**Affiliations:** Department of Sociology, Stanford University, Stanford, CA, USA; Department of Global Public Health, Karolinska Institutet, Stockholm, Sweden; Department of Global Public Health, Karolinska Institutet, Stockholm, Sweden; Department of Women’s and Children’s Health, Karolinska Institutet, Stockholm, Sweden; World Values Survey Association, Stockholm, Sweden; Department of Global Public Health, Karolinska Institutet, Stockholm, Sweden; Department of Infectious Diseases, South General Hospital, Venhälsan, Stockholm, Sweden

## Abstract

**Background:**

In many countries, immigrants face higher risks of contracting and dying from COVID-19 compared with the native-born population. Moreover, their COVID-19 vaccination uptake tends to be lower. This study aimed to investigate COVID-19 vaccine hesitancy in relation to sociodemographic characteristics, COVID-19-related exposures and social values, norms and perceptions among first-generation immigrants in Sweden. Vaccine hesitancy is an important public health issue to ensure protection against vaccine-preventable mortality and morbidity.

**Methods:**

Nation-wide representative data were collected by the Migrant World Values Survey. Descriptive and multinomial multivariate analyses were performed to analyze vaccine hesitancy among 2612 men and women aged ≥16 years.

**Results:**

One-quarter of the respondents expressed some degree of vaccine hesitancy; 5% said they would definitely not vaccinate, 7% probably not, 4% did not know and 7% did not want to answer. Young age, arriving to Sweden during the large migration wave in 2015, Eastern European origin, female gender, lower education and low trust in authorities, and less perceived benefits of vaccination were all significant determinants of vaccine hesitancy.

**Conclusions:**

The results underscore the importance of trust in healthcare providers and government authorities. Additionally, the importance of providing adequate and targeted information about vaccination to groups who face the largest barriers to care, enabling informed decision-making about the benefits and risks of vaccination in relation to health risks. Given these health risks, it is crucial that government agencies and the health sector address the multiple social dimensions that shape the low vaccine uptake and, in turn, health equity.

## Introduction

Global evidence has revealed that immigrants are at disproportionate risk of COVID-19-related morbidity and mortality, including in Sweden.^1–4^ The disproportionate mortality burden of immigrants in Sweden cannot be fully explained by language barriers,^5^ occupation or work situation,^6^ living situations, differences in underlying health or a lower propensity for hospitalization.^4^ Moreover, COVID-19 vaccine uptake is lower among immigrants in many countries including Sweden.^7–13^

Vaccine hesitancy is defined as the delay in acceptance, reluctance or refusal of vaccination. It is an important factor in who chooses to vaccinate, in turn determining the risk of contracting and spreading communicable diseases.^14^ Hence, addressing vaccine hesitancy is an important public health priority.^15^

The Swedish COVID-19 strategy builds on mutual trust between the government and individuals, placing much of the responsibility of complying with disease prevention regulations on the citizens to avoid a sharp lockdown although restaurants, shops and social gatherings were sharply restricted to very small groups.^16^ A 2021 population survey showed very high compliance with COVID-19 regulations among Swedes, as over 90% reported complying with recommendations of social distancing, practicing hand hygiene, avoiding public transport and working from home.^17^ Vaccination against COVID-19 started in Sweden in December 2020 and is available free of charge on a voluntary basis, but vaccine uptake is lower among immigrants despite universal access to healthcare.^18^ In August 2021, at the time of data collection for this study, 81% of Swedes above age 16 years had been fully vaccinated, but only 68% among foreign-borns.^18^

Qualitative interviews by our team with first-generation immigrants from Somalia and Syria (the two largest immigrant groups in Sweden) indicated a desire to wait until having received proper information from a trusted source, but not necessarily to abstain from COVID-19 vaccination. While the respondents were well aware of the risks and benefits of vaccination, the digitalization of healthcare (such as online booking systems and digital healthcare appointments), lack of information in one’s own language and delayed appointments were mentioned as barriers to vaccine information.^19^

Against this background, this article aims to investigate COVID-19 vaccine hesitancy in relation to sociodemographic characteristics, values and perceptions, as well as COVID-19-related exposures among first-generation immigrants in Sweden, using an original survey. To our knowledge, our study is the first to quantify COVID-19 vaccine hesitancy among immigrants in Sweden.

## Understanding vaccine hesitancy

We based our analysis of vaccine hesitancy on the framework developed by the SAGE Working Group on Vaccine Hesitancy.^14^ The majority of populations globally accept or embrace vaccination, but some refuse certain vaccines, delay vaccination or tolerate being immunized but feel uncertain. Vaccine hesitancy is situated on a scale between having a strong desire to get vaccinated and a total refusal to have any vaccinations. But demand and hesitancy are not completely opposite, as individuals or communities might fully accept vaccination without explicitly asking for it.

The ‘3 Cs’ vaccine hesitancy model highlights three categories: complacency, convenience and confidence. Complacency results from a low perceived risk of vaccine-preventable diseases, other life or health priorities seeming more important, and the perceived or real ability to take action to vaccinate. Convenience is based on access and availability, ability to understand and appeal of vaccination services. Confidence reflects trust in the effectiveness and safety of vaccines and the system that delivers them.^14^

As illustrated by the ‘3 Cs’ model, vaccine hesitancy is an important component of health behaviour and reflects a complex decision-making process. According to past research, it may be related to numerous factors, including language and health literacy; education and information; consulting patterns and access to health services; trust in healthcare providers and government authorities; perceived risks and benefits of vaccination and the values, perceptions and socioeconomic status of individuals and groups.^7^^,^^12–15^^,^^19–23^ Hence, vaccine hesitancy does not simply reflect individual choices, but also the larger social, economic, political and historical context where they take place.^11^ Understanding vaccine hesitancy is thus critical to health equity, and more research is needed about the extent to which and why certain populations exhibit vaccine hesitancy.

## Methods

### Data collection

We collected cross-sectional survey data of first-generation immigrants via the Migrant World Values Survey (mWVS) in April–May (wave 1) and August–September (wave 2) 2021.

The World Values Survey (WVS) is a non-governmental non-profit association that has surveyed norms and values in population-representative samples in >100 countries over the past 40 years (www.worldvaluessurvey.org). The mWVS survey instrument was adapted by the Public Health Agency of Sweden in collaboration with the WVS Association and Karolinska Institutet. It was developed in Swedish and translated into seven additional languages: Arabic, Dari, Somali, Tigrinya, Turkish and English.

The mWVS sample was based on the population register of Sweden, drawing a representative sample based on the country of origin, age and gender structure. In the first wave, the mWVS sample was complemented by adding an Internet-based random sample collected by the analytics company Novus (446 of 2732 respondents or 16% of the total sample). The Novus sample is not fully representative; it included older, more highly educated persons who had lived in Sweden longer than the mWVS sample. Wave 2 used the mWVS sampling strategy only. We treated the two waves and two modes of data collection as one sample due to the short time period between the two. See Supplementary tables A1 and A2 for descriptive statistics for each wave, and Supplementary tables B1 and B2 for sensitivity analyses separating the two waves. Separating between the mWVS and Novus samples in the first wave was not possible with the dataset available to us.

The survey was administered combining computer-assisted personal interviews (CAPI) and web interviews (CAWI). CAPI was carried out in collaboration with educational programs, such as ‘Swedish for Immigrants’ language courses, and civil society organizations. Employees of mWVS born in several of the included countries participated in translation work and data collection. The response rates were 36.5% in mWVS wave 1, 16.3% in Novus wave 1 and 47.2% in mWVS wave 2.

Individuals aged 16 years and above were sampled as this was the age recommended for COVID-19 vaccination by the Public Health Agency of Sweden at the time of data collection. Minors aged 16–17 years could decide to vaccinate without parental consent if the administering healthcare personnel considered them sufficiently mature.

From the full sample of 2732 respondents, we removed 104 second-generation immigrants, one respondent with an unknown country of birth, one without a valid birth year, five without a valid year of arrival to Sweden and nine without a valid response on gender using list-wise deletion. In total, 120 observations (4%) of the sample were removed, resulting in an analytical sample of 2612 respondents.

### Variables

The main dependent variable ‘vaccine hesitancy’ was captured by the question ‘Will you be vaccinated against COVID-19 when offered the opportunity?’ We coded this item as a nominal scale variable with five separate values (yes or already vaccinated, no probably not, no definitely not, don’t know, don’t want to answer).

An additional dependent variable captured ‘would recommend others to vaccinate’ measured by the question ‘Do you encourage people in your surroundings to accept the COVID-19 vaccine when offered vaccination?’ (yes, no probably not, no definitely not).

We did not dichotomize these two dependent variables to enable an analysis of the nuances in vaccine hesitancy.

We included the following sociodemographic variables: ‘age group’, ‘country of birth’, ‘year of arrival’ to Sweden, ‘gender’, the ‘highest level of education’, ‘relationship status’, ‘type of place of residence’ and whether the respondent was ‘living with a senior’ above age 65 years.

We also include variables on how important respondents thought it would be to ‘vaccinate for their own health’ or ‘vaccinate to protect others’, ‘trust in Swedish authorities’ and whether the respondent reported affirmatively to the following items: ‘past COVID-19 infection’, ‘worried about getting seriously ill’ and ‘worried about a close relative getting seriously ill’.

We descriptively study two multichoice variables related to the respondents’ motivation for COVID-19 vaccination: ‘reasons to vaccinate’ (protect own health, protect others from disease, return to normal) and ‘reasons to not vaccinate’ (does not believe will get seriously ill, wants more information, worried about side effects, wants to wait).

Respondents could answer ‘don’t know’ or ‘don’t want to answer’ to all survey questions. Since non-responses might reflect hesitancy towards vaccination and a reluctance to answer survey items due to, e.g. a lack of trust in authorities,^14^ we kept these response categories in our models.

### Statistical analysis

The statistical analysis started with exploratory and descriptive analysis of vaccine hesitancy. In our main analysis, we used multinomial logistic regression in which estimates are reported as relative risk ratios (RRR). Multinomial regressions are appropriate when the outcome variable is at the nominal scale, i.e. when there are multiple values in the outcome variable that do not have a natural order or ranking. In the first main model, we estimated the sociodemographic determinants of vaccine hesitancy. Next, we added COVID-19-related factors separately while controlling for sociodemographic characteristics. The two multichoice variables capturing motivations to vaccinate (or not) were omitted from the multivariate models due to insufficient variation.

### Ethical considerations

Ethical approval for this study was obtained from the Swedish Ethical Review Authority (reference number 2021–03706). Oral and written information about the study was provided through informed consent forms. Confidentiality and anonymity in the data analysis were ensured.

## Results

### Descriptive statistics

Table 1 displays descriptive statistics of the sample population. Among the 2612 individuals, more than three-quarters intended to vaccinate or had already been vaccinated against COVID-19. Among the 23% who were hesitant towards vaccination, 7% reported that they probably did not want to vaccinate and 5% were firmly against it. Those who were not sure amounted to 4%, and 7% did not want to answer the question.

**Table 1:** Descriptive statistics of the study population (*n* = 2612)

Variable and values	*n*	%
Vaccine hesitancy		
Yes or already vaccinated	2011	77
No probably not	186	7
No definitely not	139	5
Don’t know	94	4
Don’t want to answer	182	7
Would recommend others to vaccinate		
Yes	1864	71
No probably not	110	4
No definitely not	147	6
Don’t know	208	8
Don’t want to answer	283	11
Age		
Under 25	319	12
25–34	738	28
35–44	742	28
45–54	471	18
55+	342	13
Country of birth		
Middle East and North Africa	1373	53
Sub-Saharan Africa	760	29
Eastern Europe	107	4
Western Europe, North America, Australia or New Zealand	92	4
South America	106	4
Asia	174	7
Year of arrival to Sweden		
2007 or earlier	586	22
2008–14	606	23
2015	553	21
2016–20	867	33
Gender		
Female	1288	49
Male	1324	51
Highest level of education		
Primary or lower	234	9
Secondary	948	36
Tertiary	652	25
Don’t know/no answer	778	30
Relationship status		
Single	535	21
Partner	91	4
Cohabiting or married	1645	63
Divorced, separated or widowed	287	11
Don’t know/no answer	54	2
Type of place of residence		
Countryside or smaller city	330	13
City	1080	41
Big city	928	36
Don’t know/no answer	274	11
Living with senior (65 years or older)		
No	2317	89
Yes	162	6
Don’t know/no answer	133	5
Trust in Swedish authorities		
Very low	212	8
Somewhat low	266	10
Neither low nor high	336	13
Somewhat high	589	23
Very high	386	15
Don’t know	155	6
Don’t want to answer	677	26
Importance of COVID 19-vaccination for own health		
Not important at all	144	6
Not very important	141	5
Fairly important	515	20
Very important	1294	50
Don’t know	226	9
Don’t want to answer	292	11
Importance of COVID 19-vaccination to protect others		
Not important at all	97	4
Not very important	76	3
Fairly important	474	18
Very important	1513	58
Don’t know	181	7
Don’t want to answer	271	10
Past COVID-19 infection		
No	1428	55
Yes (definitely or maybe)	543	21
Don’t know	317	12
Don’t want to answer	324	12
Worried about becoming seriously ill in COVID-19		
Not at all	500	19
To some extent	821	31
Fairly much	368	14
Very much	298	11
Don’t know	148	6
Don’t want to answer	477	18
Worried about someone close/a relative becoming seriously ill in COVID-19		
Not at all	228	9
To some extent	643	25
Fairly much	521	20
Very much	615	24
Don’t know	123	5
Don’t want to answer	482	19
Total	2612	100

Moreover, 71% would recommend others to vaccinate. One in 10 would probably (4%) or definitely (6%) not do so, while almost one-fifth did not want to answer or did not know.

**Figure 1:**
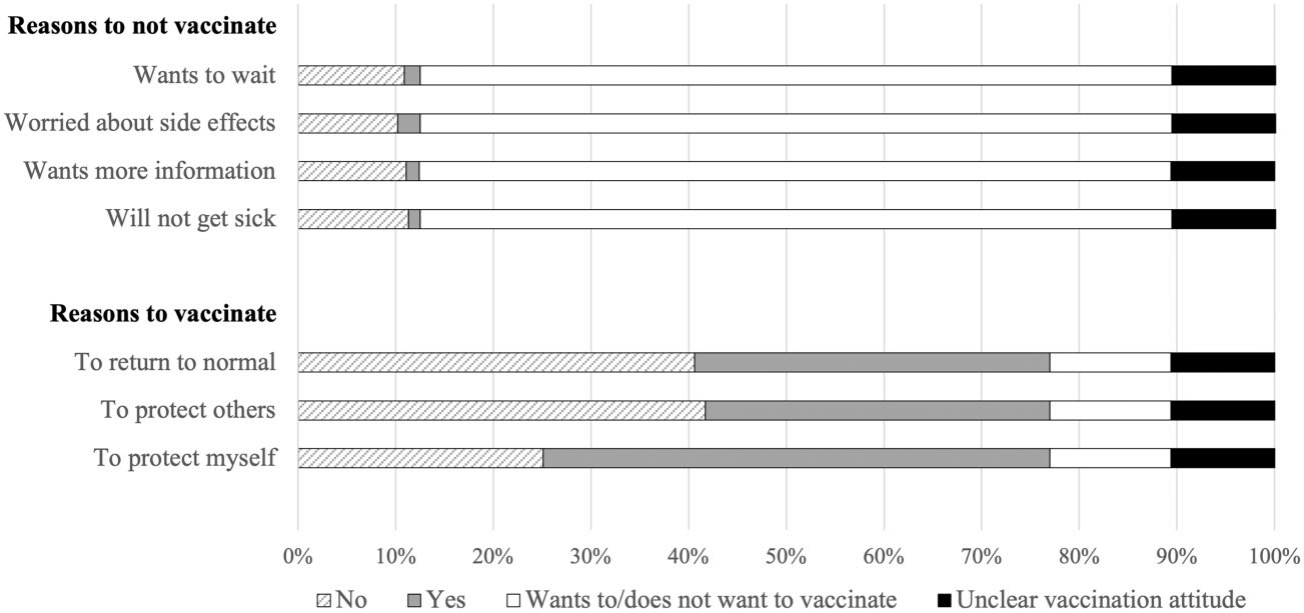
Motivations for and against vaccination *Note*: Only those that reported that they did not want to vaccinate were asked about motivations against vaccination (and vice versa). The white segment of the bars represents those who were not asked. The black segment of the bars illustrates those who did not give a valid answer on the item measuring vaccine hesitancy.

Figure 1 displays descriptive statistics of motivations for and against vaccination. Out of those who were hesitant towards vaccination, only 1 -2% stated they did not want to vaccinate because of a belief they would not get seriously ill in COVID-19, wanting more information, worrying about side effects, or wanting to wait. Among those who were positive towards vaccination or already vaccinated, more than half reported protection of themselves as the main motivation to vaccinate, whereas around one-third reported protecting others and returning to normal as the main reasons.

Notably, around three-quarters believed vaccination is fairly or very important to protect themselves or others. More than half were positive towards vaccination to protect their own health, while more than one-third believed it is important to protect others’ health and to return to normal.

Almost half of the respondents had somewhat or very high trust in Swedish authorities. Only one-quarter were fairly or very worried about becoming seriously ill in COVID-19. Almost half were fairly or very worried about a relative or close one becoming seriously ill in COVID-19.

The descriptive statistics thus suggest that vaccine hesitancy was not a dominant feature and not the main reason for non-vaccination among these first-generation migrant participants.

### Sociodemographic determinants of vaccine hesitancy

Next, we explored how vaccine hesitancy varied within subgroups of the sampled population. The multinomial multivariate regression in table 2 estimated the sociodemographic determinants of vaccine hesitancy, in comparison to those who wanted to vaccinate or had already been vaccinated.

**Table 2:** Vaccine hesitancy in relation to sociodemographic factors

Will get vaccinated	No probably not (*n* = 186)		No definitely not (*n* = 139)		Don’t know (*n* = 94)		Don’t want to answer (*n* = 192)	
	RRR	CIs	RRR	CIs	RRR	CIs	RRR	CIs
Age group (ref. 35–44)								
Under 25	0.93	(0.52–1.67)	2.06*	(1.07–3.95)	1.98	(0.99–3.99)	0.81	(0.37–1.79)
25–34	1.04	(0.71–1.52)	1.69*	(1.05–2.72)	0.83	(0.47–1.46)	1.04	(0.58–1.86)
45–54	0.49**	(0.29–0.82)	0.94	(0.54–1.65)	0.38*	(0.18–0.83)	0.85	(0.41–1.75)
55+	0.30**	(0.14–0.63)	0.46	(0.20–1.03)	0.30**	(0.12–0.74)	0.89	(0.39–2.03)
Country of birth (ref. Western Europe, North America, Australia or New Zealand)								
Middle East and North Africa	2.57	(0.61–10.92)	2.62	(0.61–11.20)	0.96	(0.32–2.88)	0.52	(0.13–2.15)
Sub-Saharan Africa	2.45	(0.57–10.55)	2.43	(0.56–10.59)	0.84	(0.27–2.63)	1.14	(0.28–4.68)
Eastern Europe	6.18*	(1.31–29.10)	10.57**	(2.28–48.94)	2.15	(0.56–8.29)	1.10	(0.17–7.03)
South America	2.08	(0.38–11.30)	2.39	(0.44–12.95)	0.71	(0.15–3.43)	0.44	(0.04–4.43)
Asia	1.26	(0.24–6.53)	1.73	(0.34–8.75)	0.49	(0.11–2.13)	1.09	(0.19–6.18)
Year of arrival (ref. 2007 or earlier)								
2008–14	1.15	(0.63–2.12)	0.68	(0.37–1.26)	0.49	(0.23–1.06)	1.48	(0.50–4.44)
2015	1.11	(0.59–2.08)	0.41*	(0.20–0.82)	0.41*	(0.19–0.93)	1.60	(0.53–4.84)
2016–20	1.20	(0.67–2.16)	0.75	(0.42–1.34)	0.60	(0.29–1.21)	1.77	(0.62–5.06)
Gender (ref. woman)								
Male	0.67*	(0.49–0.92)	0.83	(0.58–1.18)	0.83	(0.54–1.27)	0.94	(0.60–1.46)
Highest level of education (ref. secondary)								
Primary or lower	0.75	(0.40–1.41)	2.25**	(1.33–3.81)	2.30*	(1.13–4.68)	1.13	(0.33–3.91)
Tertiary	0.68	(0.43–1.09)	0.71	(0.43–1.19)	0.93	(0.48–1.80)	1.53	(0.50–4.67)
Don’t know/no answer	1.35	(0.94–1.94)	1.04	(0.65–1.66)	2.45**	(1.43–4.19)	11.16***	(5.52–22.58)
Relationship status (ref. single)								
Partner	1.27	(0.56–2.89)	0.29	(0.07–1.25)	0.17	(0.02–1.31)	0.64	(0.15–2.64)
Cohabiting or married	1.06	(0.68–1.65)	0.79	(0.48–1.28)	0.66	(0.38–1.17)	0.80	(0.44–1.47)
Divorced, separated or widowed	1.42	(0.78–2.60)	1.29	(0.67–2.48)	0.93	(0.41–2.13)	0.45	(0.17–1.18)
Don’t know/no answer	0.95	(0.20–4.44)	1.31	(0.28–6.18)	0.70	(0.09–5.63)	2.03	(0.63–6.55)
Type of place of residence (ref. city)								
Countryside or smaller city	1.14	(0.71–1.83)	1.27	(0.75–2.13)	1.54	(0.83–2.89)	1.58	(0.70–3.57)
Big city	0.98	(0.68–1.41)	1.04	(0.69–1.57)	1.34	(0.82–2.19)	1.42	(0.75–2.70)
Don’t know/no answer	1.74*	(1.00–3.00)	0.86	(0.38–1.99)	0.69	(0.23–2.05)	13.96***	(7.69–25.33)
Living with senior (ref. no)								
Yes	0.98	(0.46–2.09)	0.87	(0.39–1.98)	1.60	(0.73–3.51)	1.09	(0.43–2.76)
Don’t know/no answer	1.28	(0.47–3.49)	0.39	(0.05–3.00)	1.06	(0.24–4.76)	10.33***	(5.46–19.54)
Constant	0.04***	(0.01–0.21)	0.04***	(0.01–0.19)	0.09***	(0.02–0.35)	0.01***	(0.00–0.04)

* *P* < 0.05;

** *P* < 0.01;

*** *P* < 0.001.

RRR, relative risk ratio; CIs, confidence intervals; ref., reference; base category, wants to vaccinate or already vaccinated; *n* = 2612.

Individuals younger than 35 years were twice as likely to refuse vaccination, whereas older participants had a significantly lower risk of refusing the COVID-19 vaccine (RRR = 0.49 in ages 45–54, RRR = 0.30 in ages 55+), compared with those aged 35–44 years. Respondents over 45 years of age were also less likely to feel uncertain about whether to vaccinate or not (RRR = 0.38 in ages 45–54, RRR = 0.30 in those aged 55+).

Respondents born in Eastern Europe were substantially more likely to probably (RRR = 6.18) or definitely (RRR = 10.57) want to abstain from vaccination as compared with being born in Western Europe, North America, Australia or New Zealand. Results were similar when disaggregating by country (available upon request).

Those who arrived in 2015 had a significantly lower risk of being completely opposed (RRR = 0.41) or unsure (RRR = 0.41). This year was characterized by a substantially higher number of refugees arriving in Sweden, a large share being young men from Afghanistan or families fleeing the war in Syria. Males were at lower risk of probably not wanting to vaccinate compared with women (RRR = 0.67).

Respondents with primary or lower levels of education had more than twice the risks of definitely not wanting to vaccinate (RRR = 2.25) or being unsure (RRR = 2.30), compared with those with secondary education.

Being unsure or refusing to answer the question about vaccine hesitancy was significantly related to non-responses for education, residence and living with a senior. In other words, some individuals chose to skip several questions.

Other sociodemographic characteristics were not statistically different for the risk of vaccine hesitancy when compared with the reference categories.

### Values, perceptions and COVID-19-related factors

Table 3 includes items measuring social values and perceptions as well as COVID-19-related factors analysed one by one in separate models, controlling for the sociodemographic factors included in table 2 (a model combining all factors presented in table 3 is available in Supplementary table SB). Again, RRR for vaccine hesitancy are displayed in reference to those who wanted to vaccinate or had already been vaccinated.

**Table 3:** Vaccine hesitancy in relation to COVID-19-related factors

Will get vaccinated	No probably not (*n* = 186)		No definitely not (*n* = 139)		Don’t know (*n* = 94)		Don’t want to answer (*n* = 192)	
	RRR	CIs	RRR	CIs	RRR	CIs	RRR	CIs
Trust in Swedish authorities (ref. neither big or little)								
Very little	0.79	(0.37–1.68)	1.14	(0.60–2.17)	1.34	(0.59–3.06)	1.44	(0.19–10.94)
Quite a bit	0.79	(0.40–1.56)	0.69	(0.35–1.36)	1.04	(0.46–2.36)	1.14	(0.15–8.80)
Somewhat high	0.76	(0.44–1.34)	0.52*	(0.29–0.94)	0.55	(0.25–1.21)	1.03	(0.18–5.81)
Very high	0.65	(0.34–1.25)	0.33**	(0.16–0.69)	0.42	(0.16–1.08)	1.77	(0.31–10.24)
Don’t know	2.51**	(1.32–4.77)	1.17	(0.55–2.46)	0.76	(0.25–2.28)	1.87	(0.28–12.72)
Don’t want to answer	1.30	(0.61–2.78)	1.02	(0.42–2.50)	1.08	(0.39–2.96)	16.72**	(2.95–94.83)
Constant	0.06***	(0.02–0.18)	0.09***	(0.03–0.27)	0.05***	(0.01–0.20)	0.00***	(0.00–0.04)
Importance of vaccination for own health (ref. not important at all)								
Not very important	1.84	(0.72–4.72)	5.97***	(2.60–13.74)	0.50	(0.23–1.08)	2.76	(0.53–14.31)
Fairly important	0.55	(0.24–1.23)	0.80	(0.36–1.77)	0.04***	(0.02–0.10)	0.59	(0.14–2.47)
Very important	0.18***	(0.08–0.40)	0.06***	(0.02–0.17)	0.02***	(0.01–0.04)	0.12**	(0.03–0.60)
Don’t know	11.30***	(5.19–24.62)	3.96**	(1.70–9.23)	0.28**	(0.13–0.64)	2.32	(0.52–10.27)
Don’t want to answer	2.03	(0.83–4.96)	2.70*	(1.07–6.82)	0.68	(0.33–1.41)	16.75***	(4.56–61.51)
Constant	0.06***	(0.01–0.22)	0.06***	(0.02–0.23)	0.43	(0.10–1.80)	0.01***	(0.00–0.10)
Importance of vaccination to protect others (ref. not important at all)								
Not very important	17.71**	(2.08–150.88)	12.22***	(3.75–39.80)	1.27	(0.51–3.15)	2.13	(0.31–14.49)
Fairly important	8.29*	(1.11–61.86)	2.55	(0.87–7.48)	0.18***	(0.09–0.39)	0.50	(0.12–2.09)
Very important	1.86	(0.25–13.89)	0.19**	(0.06–0.60)	0.03***	(0.01–0.07)	0.13**	(0.03–0.54)
Don’t know	94.46***	(12.47–715.25)	12.56***	(4.03–39.14)	1.06	(0.47–2.41)	1.57	(0.33–7.44)
Don’t want to answer	14.31*	(1.79–114.29)	6.18**	(1.83–20.89)	0.78	(0.33–1.85)	15.11***	(3.93–58.05)
Constant	0.01***	(0.00–0.08)	0.05***	(0.01–0.20)	0.25	(0.06–1.01)	0.02***	(0.00–0.15)
Past COVID-19 infection (ref. no)								
Yes (definitely or maybe)	1.04	(0.70–1.55)	1.42	(0.93–2.18)	1.32	(0.78–2.24)	0.54	(0.18–1.62)
Don’t know	1.42	(0.90–2.23)	1.08	(0.61–1.92)	1.12	(0.56–2.25)	0.75	(0.22–2.59)
Don’t want to answer	1.22	(0.67–2.21)	1.45	(0.72–2.95)	1.50	(0.72–3.12)	15.78***	(8.49–29.34)
Constant	0.04***	(0.02–0.12)	0.06***	(0.02–0.16)	0.03***	(0.01–0.13)	0.00***	(0.00–0.02)
Worried about becoming seriously ill in COVID-19 (ref. not at all)								
To some extent	0.96	(0.61–1.52)	0.50**	(0.32–0.79)	0.17***	(0.09–0.32)	0.34	(0.10–1.13)
Fairly much	0.57	(0.30–1.05)	0.22***	(0.10–0.46)	0.26***	(0.12–0.55)	1.23	(0.41–3.70)
Very much	0.39*	(0.18–0.84)	0.35**	(0.18–0.68)	0.17***	(0.06–0.44)	0.41	(0.08–2.10)
Don’t know	2.76***	(1.55–4.91)	0.77	(0.35–1.67)	0.64	(0.27–1.52)	1.37	(0.34–5.53)
Don’t want to answer	1.23	(0.66–2.27)	0.79	(0.38–1.65)	0.47*	(0.22–0.99)	7.33***	(2.58–20.85)
Constant	0.05***	(0.02–0.15)	0.11***	(0.04–0.31)	0.10***	(0.03–0.37)	0.01***	(0.00–0.06)
Worried about someone close/a relative becoming seriously ill in COVID-19 (ref. not at all)								
To some extent	0.73	(0.39–1.34)	0.62	(0.34–1.14)	0.31***	(0.17–0.59)	0.22*	(0.06–0.88)
Fairly much	0.63	(0.33–1.19)	0.50*	(0.26–0.95)	0.13***	(0.06–0.30)	0.28	(0.08–1.03)
Very much	0.45*	(0.24–0.88)	0.26***	(0.13–0.53)	0.16***	(0.08–0.34)	0.34	(0.10–1.17)
Don’t know	3.03**	(1.50–6.11)	1.48	(0.66–3.29)	0.42	(0.15–1.22)	0.52	(0.09–3.06)
Don’t want to answer	1.08	(0.53–2.20)	0.84	(0.37–1.89)	0.37*	(0.17–0.81)	3.92*	(1.26–12.22)
Constant	0.07***	(0.02–0.21)	0.11***	(0.04–0.33)	0.14**	(0.04–0.53)	0.02***	(0.00–0.11)

*n* = 2612; relative risks are displayed net of SES variables included in table 2.

* *P* < 0.05;

** *P* < 0.01;

*** *P* < 0.001.

RRR, relative risk ratio; CIs, confidence intervals; ref., reference; base category, wants to vaccinate or already vaccinated.

Those who had somewhat or very high trust in Swedish authorities had a significantly lower risk of vaccine hesitancy (RRR = 0.52 or 0.33, respectively). Those who did not know whether they trusted Swedish authorities had more than twice as high a risk of probably not wanting to vaccinate (RRR = 2.51).

Those who believed it is fairly or very important to vaccinate to protect the health of oneself or others were overall significantly less likely to express various degrees of vaccine hesitancy (RRR = 0.02–8.29), while those who did not believe it is important (RRR = 5.97–17.71), did not know (RRR = 3.96–94.46) or did not want to answer (RRR = 2.70–14.31) were generally at higher risk of vaccine hesitancy. Thus, perceptions of the benefits of vaccination seem to correlate with the intention to vaccinate.

Past COVID-19 infection was not significantly related to vaccine hesitancy, except in refusal to answer both questions.

Compared with not being worried about either themselves, someone close or a relative becoming seriously ill in COVID-19, those who were worried were significantly less at risk of not wanting to vaccinate, while those who did not know were at higher risk. Again, this point towards risk assessment and perceived benefits of vaccination as relevant determinants of vaccine hesitancy.

Regression models of the alternative dependent variable measuring whether one would recommend others to vaccinate against COVID-19 are available in Supplementary tables SC and SD.

## Discussion

This study showed that more than three-quarters among first-generation immigrants in Sweden were positive towards COVID-19 vaccination or had already been vaccinated, indicating that vaccine hesitancy was not a dominant feature among immigrants’ lower uptake of vaccination during the summer of 2021.

Qualitative interviews with Somali and Syrian immigrants to Sweden have shown that immigrants were not very opposed to but rather wanted more information before vaccination, preferably from a trusted peer (e.g. religious leader or compatriot) or doctor.^19^ This is consistent with the results in this study since many respondents did not know whether they wanted to vaccinate or recommend others to vaccinate, indicating a lack of sufficient information. According to the ‘3 Cs’ model, this suggests that the foremost obstacle to COVID-19 vaccination is convenience; access and availability, ability to understand and appeal of vaccination services. While individuals may be willing, structural barriers can block their vaccination uptake.^14^ Our findings are valuable in that they could guide the response during future pandemics and also improve equal access to care, since information is vital to making choices regarding health and healthcare.

Some important individual-level characteristics distinguish the respondents who expressed vaccine reluctance or refusal, related to age, year of arrival, country of birth, gender and education. Age differentials have also been found in most international evidence^8^^,^^9^^,^^22^ and are likely explained by older people’s higher risk of becoming seriously ill with SARS-CoV-2, thus reflecting perceived benefits of vaccination or what the ‘3 Cs’ model describes as complacency.^14^

In 2015, a substantially higher number of refugees arrived in Sweden during a short time period, the highest migration influx per capita in Europe.^24^ Those who arrived this year expressed lower vaccine hesitancy all else equal. Though impossible to determine with the available data, one could speculate that the overall policy climate that immigrants meet upon arrival shapes their subsequent health behaviours.

Among the various countries of origin, only Eastern Europe stood out with a higher risk of vaccine hesitancy, probably reflecting a high skepticism towards authorities in general among migrants from these countries, resulting in lower COVID-19 vaccination uptake overall in Eastern Europe.^25^

Women’s higher vaccine hesitancy may be surprising since men are usually less prone to seek healthcare, but mirrors tendencies in other countries such as Australia, France, the UK and the USA (although other studies from Ireland and the UK found no gender differences).^8^^,^^9^ It might reflect worries about side effects on fecundity, or that men are more integrated in society (e.g. in the labour market) and/or more worried about income loss during illness.

Vaccine hesitancy among individuals with lower levels of education could reflect health literacy and less access to information, as corroborated by some but not all international evidence.^8^^,^^9^ Health literacy and the ability to take in information, too, represent the convenience of vaccination.

Among the respondents’ values, perceptions and COVID-19-related determinants, several factors shaped the risk of vaccine hesitancy: trust in authorities, attitudes towards the importance of vaccination to protect one’s health or the health of others, as well as worry about becoming seriously ill in COVID-19. These findings show how COVID-19 vaccine hesitancy is driven by pre-existing social trust, what people think and feel about vaccination in general and individual motivations to seek vaccination, which in turn mediates health-seeking behaviours.^9–12^^,^^21^^,^^22^

The perceived risks and benefits of vaccination, again, relate to complacency. The importance of trust echoes what the ‘3 Cs’ model describes as confidence; vaccine hesitancy is shaped by trust in the system that delivers vaccines, including the reliability and competence of the health services and health professionals.^14^ This finding highlights the need for authorities to actively engage with underserved groups of the population to build trust.

### Limitations

There are some notable limitations of our study. Since the sample was limited to first-generation immigrants no comparison could be made to the Swedish-born population. We were also unable to investigate some items in the survey questionnaire (such as respondent’s income, level of integration, visa status, state of health, experiences of discrimination and language skills) due to a large share of non-specific missing values.

The high non-response rates for some included survey items may be due to a range of factors, such as social desirability bias in reporting attitudes towards vaccination, survey fatigue/distrust and/or a genuine uncertainty or refusal to answer. Many non-response categories were statistically correlated, indicating that some respondents indeed abstained from multiple questions, but their reason for doing so remains unknown. It is also likely that those that are more vaccine hesitant are also less prone to answering surveys, thus the true level of vaccine hesitancy among immigrants in Sweden might be higher than reported here.

The mWVS samples in both waves were representative of the immigrant population in Sweden. However, as described above, the 16% wave 1 Novus sample had a predominance of older, highly educated respondents who had lived in Sweden longer (see Supplementary tables A1 and A2). These groups are more likely to accept vaccination according to our findings and may thus create a downward bias in the model estimates. Since our dataset did not distinguish between the two modes of data collection, we could not exclude the Novus sample in sensitivity analyses.

### Conclusion

Our study is the first to quantify COVID-19 vaccine hesitancy among first-generation immigrants in Sweden. The results underscore the importance of trust in healthcare providers and government authorities and the importance of providing adequate information about vaccination to groups who face the largest barriers to care—such as those with lower levels of education—in order to enable informed decision-making about the benefits and risks of vaccination in relation to health risks associated with disease. If government authorities and healthcare providers clearly address misconceptions or fears regarding vaccination and potential side effects, immigrants may be more inclined to choose vaccination in turn protecting them from preventable illness and premature death.

## Supplementary Material

ckad073_Supplementary_Data

## Data Availability

Data access for research purposes may be requested from the corresponding author.
