# Supplementary material for: COVID-19 vaccine hesitancy among first-generation immigrants living in Sweden
Source: Eur J Public Health. 2023 May 25;33(4):687–94. doi: 10.1093/eurpub/ckad073 (PMC10395762; doi:10.1093/eurpub/ckad073)
Supplement: ckad073_Supplementary_Data [file ckad073_supplementary_data.docx]

## Supplementary material

Tables A1 and A2 display descriptive statistics of the two different samples in waves 1 (n=1,390) and 2 (n=1,222).

| **Table A1** Descriptive statistics of the study population sampled in wave 1 | | |
| --- | --- | --- |
| **Variable and values** | **n** | **%** |
| **Age** |  |  |
| *Under 25* | 122 | 9% |
| *25–34* | 339 | 25% |
| *35–44* | 412 | 30% |
| *45–54* | 284 | 20% |
| *55+* | 233 | 17% |
| **Country of birth** |  |  |
| *Middle East and North Africa* | 695 | 50% |
| *Sub-Saharan Africa* | 312 | 22% |
| *Eastern Europe* | 73 | 5% |
| *Western Europe, North America, Australia, or New Zealand* | 70 | 5% |
| *South America* | 93 | 7% |
| *Asia* | 147 | 11% |
| **Year of arrival to Sweden** |  |  |
| *2007 or earlier* | 509 | 37% |
| *2008–2014* | 299 | 22% |
| *2015* | 222 | 16% |
| *2016–2020* | 360 | 26% |
| **Gender** |  |  |
| *Female* | 677 | 49% |
| *Male* | 713 | 51% |
| **Highest level of education** |  |  |
| *Primary or lower* | 78 | 6% |
| *Secondary* | 483 | 35% |
| *Tertiary* | 475 | 34% |
| *Don’t know/no answer* | 354 | 25% |
| **Relationship status** |  |  |
| *Single* | 261 | 19% |
| *Partner* | 54 | 4% |
| *Cohabiting or married* | 893 | 64% |
| *Divorced, separated, or widowed* | 160 | 12% |
| *Don’t know/no answer* | 22 | 2% |
| **Type of place of residence** |  |  |
| *Countryside or smaller city* | 145 | 10% |
| *City* | 533 | 38% |
| *Big city* | 595 | 43% |
| *Don’t know/no answer* | 117 | 8% |
| **Living with senior (65 years or older)** |  |  |
| *No* | 1,251 | 90% |
| *Yes* | 90 | 6% |
| *Don’t know/no answer* | 49 | 4% |
| **Total** | **1,390** | **100%** |

| **Table A2** Descriptive statistics of the study population sampled in wave 2 | | |
| --- | --- | --- |
| **Variable and values** | **n** | **%** |
| **Age** |  |  |
| *Under 25* | 197 | 16% |
| *25–34* | 399 | 33% |
| *35–44* | 330 | 27% |
| *45–54* | 187 | 15% |
| *55+* | 109 | 9% |
| **Country of birth** |  |  |
| *Middle East and North Africa* | 678 | 55% |
| *Sub-Saharan Africa* | 448 | 37% |
| *Eastern Europe* | 34 | 3% |
| *Western Europe, North America, Australia, or New Zealand* | 22 | 2% |
| *South America* | 13 | 1% |
| *Asia* | 27 | 2% |
| **Year of arrival to Sweden** |  |  |
| *2007 or earlier* | 77 | 6% |
| *2008–2014* | 307 | 25% |
| *2015* | 331 | 27% |
| *2016–2020* | 507 | 41% |
| **Gender** |  |  |
| *Female* | 611 | 50% |
| *Male* | 611 | 50% |
| **Highest level of education** |  |  |
| *Primary or lower* | 156 | 13% |
| *Secondary* | 465 | 38% |
| *Tertiary* | 177 | 14% |
| *Don’t know/no answer* | 424 | 35% |
| **Relationship status** |  |  |
| *Single* | 274 | 22% |
| *Partner* | 37 | 3% |
| *Cohabiting or married* | 752 | 62% |
| *Divorced, separated, or widowed* | 127 | 10% |
| *Don’t know/no answer* | 32 | 3% |
| **Type of place of residence** |  |  |
| *Countryside or smaller city* | 185 | 15% |
| *City* | 547 | 45% |
| *Big city* | 333 | 27% |
| *Don’t know/no answer* | 157 | 13% |
| **Living with senior (65 years or older)** |  |  |
| *No* | 1,066 | 87% |
| *Yes* | 72 | 6% |
| *Don’t know/no answer* | 84 | 7% |
| **Total** | **1,222** | **100%** |

Tables B1 and B2 report differences between the two waves in the sociodemographic determinants of vaccine hesitancy (n=1,390 in wave 1 and 1,222 in wave 2).

In both waves, younger individuals tended to be more opposed to or unsure about vaccination. The previously reported vaccine hesitancy among Eastern Europeans as well as gender and educational differences were driven by the first wave.

In the first wave only, respondents from the Middle East and North Africa were significantly less likely to not want to answer, and immigrants who arrived in 2016 or later had a significantly higher risk of probably not wanting to vaccinate. Those who were cohabiting, married, divorced, separated, or widowed were less at risk of not wanting to answer about their vaccine attitude. Residents in the countryside and smaller cities were more likely to refuse to answer.

In the second wave only, those residing in the countryside, smaller or bigger cities were at significantly higher risk of being completely opposed to vaccination compared to those in medium-sized cities. Respondents who arrived in Sweden in 2008 or later were less likely to completely oppose or be unsure about their attitude to vaccination, compared to the group that is arguably most integrated: those who had lived in Sweden since 2007 or earlier.

In other words, perceptions may have shifted towards more acceptance of COVID-19 vaccination in some sub-groups between April–May and August–September 2021. However, the different results between the two waves should be interpreted with caution as the data are repeated cross-sectional – not longitudinal – collected using different sampling procedures at relatively short intervals.

| **Table B1** Vaccine hesitancy: Wave 1 | | | | | | | | |  |
| --- | --- | --- | --- | --- | --- | --- | --- | --- | --- |
| **Will get vaccinated:** | **No probably not**  **(n=135)** | | **No definitely not**  **(n=100)** | | **Don't know**  **(n=70)** | | **Don't want to answer**  **(n=82)** | |  |
|  |  |  |  |  |  |  |  |  |  |
|  | RRR | CIs | RRR | CIs | RRR | CIs | RRR | CIs |  |
| **Age group (ref. 35–44)** |  |  |  |  |  |  |  |  |  |
| *Under 25* | 1.53 | (0.73-3.22) | 2.40* | (1.04-5.54) | 1.60 | (0.65-3.95) | 0.64 | (0.19-2.10) |  |
| *25–34* | 1.07 | (0.67-1.72) | 1.40 | (0.79-2.49) | 0.66 | (0.33-1.31) | 1.36 | (0.59-3.12) |  |
| *45–54* | 0.53* | (0.29-0.95) | 0.93 | (0.49-1.76) | 0.41* | (0.18-0.96) | 0.99 | (0.35-2.82) |  |
| *55+* | 0.35* | (0.15-0.80) | 0.47 | (0.19-1.14) | 0.32* | (0.12-0.86) | 0.75 | (0.18-3.15) |  |
| **Country of birth (ref. Western Europe, North America, Australia or New Zealand)** |  |  |  |  |  |  |  |  |  |
| *Middle East and North Africa* | 5.70e+06 | (0.00-.) | 5.36 | (0.71-40.67) | 1.23 | (0.35-4.36) | 0.19* | (0.04-0.95) |  |
| *Sub-Saharan Africa* | 6.54e+06 | (0.00-.) | 6.32 | (0.81-49.36) | 1.28 | (0.34-4.87) | 0.74 | (0.15-3.73) |  |
| *Eastern Europe* | 1.06e+07 | (0.00-.) | 13.31* | (1.60-110.86) | 2.40 | (0.51-11.20) | 0.27 | (0.03-2.95) |  |
| *South America* | 4.32e+06 | (0.00-.) | 2.39 | (0.24-24.20) | 0.52 | (0.08-3.37) | 0.00 | (0.00-.) |  |
| *Asia* | 2.16e+06 | (0.00-.) | 2.20 | (0.25-19.41) | 0.44 | (0.08-2.37) | 0.60 | (0.10-3.74) |  |
| **Year of arrival (ref. 2007 or earlier)** |  |  |  |  |  |  |  |  |  |
| *2008–2014* | 1.59 | (0.81-3.13) | 0.94 | (0.47-1.87) | 0.52 | (0.21-1.29) | 1.68 | (0.42-6.67) |  |
| *2015* | 1.88 | (0.91-3.89) | 0.67 | (0.29-1.54) | 0.85 | (0.34-2.11) | 3.98 | (0.99-15.94) |  |
| *2016–2020* | 2.06* | (1.06-3.99) | 1.33 | (0.68-2.59) | 1.11 | (0.51-2.45) | 2.19 | (0.60-8.06) |  |
| Gender (ref. woman) |  |  |  |  |  |  |  |  |  |
| *Male* | 0.51** | (0.33-0.77) | 0.56* | (0.35-0.90) | 0.75 | (0.44-1.30) | 0.33** | (0.16-0.67) |  |
| **Highest level of education (ref. secondary)** |  |  |  |  |  |  |  |  |  |
| *Primary or lower* | 1.28 | (0.57-2.92) | 3.27** | (1.59-6.75) | 4.60*** | (1.86-11.35) | 2.15 | (0.48-9.67) |  |
| *Tertiary* | 0.67 | (0.39-1.14) | 0.70 | (0.40-1.25) | 1.00 | (0.48-2.11) | 1.00 | (0.27-3.76) |  |
| *Don't know/no answer* | 1.58* | (1.00-2.51) | 0.88 | (0.49-1.59) | 2.63** | (1.34-5.17) | 6.64*** | (2.72-16.20) |  |
| **Relationship status (ref. single)** |  |  |  |  |  |  |  |  |  |
| *Partner* | 2.23 | (0.83-6.00) | 0.51 | (0.11-2.36) | 0.31 | (0.04-2.43) | 0.88 | (0.17-4.53) |  |
| *Cohabiting or married* | 1.18 | (0.65-2.13) | 0.76 | (0.41-1.42) | 0.51 | (0.25-1.02) | 0.38* | (0.17-0.87) |  |
| *Divorced, separated or widowed* | 1.60 | (0.73-3.52) | 1.38 | (0.61-3.14) | 0.98 | (0.38-2.54) | 0.04** | (0.00-0.43) |  |
| *Don't know/no answer* | 0.68 | (0.07-6.16) | 0.00 | (0.00-.) | 0.61 | (0.06-6.04) | 1.54 | (0.25-9.56) |  |
| **Type of place of residence (ref. city)** |  |  |  |  |  |  |  |  |  |
| *Countryside or smaller city* | 0.86 | (0.44-1.65) | 0.72 | (0.34-1.53) | 2.03 | (0.94-4.40) | 3.48* | (1.13-10.75) |  |
| *Big city* | 0.91 | (0.59-1.39) | 0.72 | (0.44-1.16) | 1.22 | (0.68-2.21) | 2.03 | (0.81-5.07) |  |
| *Don't know/no answer* | 1.55 | (0.77-3.13) | 0.82 | (0.31-2.14) | 0.99 | (0.31-3.15) | 17.70*** | (6.77-46.29) |  |
| **Living with senior (ref. no)** |  |  |  |  |  |  |  |  |  |
| *Yes* | 1.05 | (0.43-2.60) | 0.79 | (0.27-2.33) | 2.01 | (0.82-4.92) | 0.63 | (0.12-3.29) |  |
| *Don't know/no answer* | 1.59 | (0.38-6.64) | 0.83 | (0.09-7.41) | 0.72 | (0.08-6.48) | 19.79*** | (6.28-62.41) |  |
| **Constant** | 0.00 | (0.00-.) | 0.04** | (0.00-0.33) | 0.10** | (0.02-0.50) | 0.04** | (0.00-0.31) |  |
| ∗Significant at p < 0.05; ∗∗p < 0.01; ∗∗∗p < 0.001; RRR=relative risk ratio; CIs=confidence intervals; ref.=reference; base category=wants to vaccinate or already vaccinated, n=,1390 | | | | | | | | |  |

| **Table B2** Vaccine hesitancy: Wave 2 | | | | | | | | |  |
| --- | --- | --- | --- | --- | --- | --- | --- | --- | --- |
| **Will get vaccinated:** | **No probably not**  **(n=51)** | | **No definitely not**  **(n=39)** | | **Don't know**  **(n=24)** | | **Don't want to answer**  **(n=100)** | |  |
|  |  |  |  |  |  |  |  |  |  |
|  | RRR | CIs | RRR | CIs | RRR | CIs | RRR | CIs |  |
| **Age group (ref. 35–44)** |  |  |  |  |  |  |  |  |  |
| *Under 25* | 0.71 | (0.25-2.01) | 4.33* | (1.19-15.66) | 8.83** | (1.99-39.23) | 1.24 | (0.36-4.27) |  |
| *25–34* | 1.16 | (0.58-2.34) | 3.92* | (1.34-11.42) | 2.37 | (0.66-8.52) | 0.86 | (0.36-2.04) |  |
| *45–54* | 0.48 | (0.15-1.51) | 1.44 | (0.36-5.67) | 0.36 | (0.04-3.41) | 0.75 | (0.26-2.17) |  |
| *55+* | 0.22 | (0.03-1.82) | 0.40 | (0.04-4.04) | 0.00 | (0.00-.) | 0.93 | (0.30-2.92) |  |
| **Country of birth (ref. Western Europe, North America, Australia or New Zealand)** |  |  |  |  |  |  |  |  |  |
| *Middle East and North Africa* | 0.44 | (0.08-2.35) | 0.86 | (0.09-8.08) | 0.27 | (0.02-3.14) | 1.09 | (0.10-11.35) |  |
| *Sub-Saharan Africa* | 0.51 | (0.10-2.66) | 0.85 | (0.09-7.91) | 0.24 | (0.02-2.73) | 1.53 | (0.15-15.69) |  |
| *Eastern Europe* | 1.11 | (0.13-9.82) | 7.75 | (0.68-88.42) | 0.53 | (0.02-12.65) | 2.99 | (0.15-58.19) |  |
| *South America* | 0.00 | (0.00-.) | 9.79 | (0.62-155.40) | 4.43 | (0.18-108.40) | 1.35 | (0.05-38.15) |  |
| *Asia* | 0.00 | (0.00-.) | 0.64 | (0.03-13.35) | 0.45 | (0.02-10.34) | 0.31 | (0.01-10.63) |  |
| **Year of arrival (ref. 2007 or earlier)** |  |  |  |  |  |  |  |  |  |
| *2008–2014* | 0.77 | (0.15-4.03) | 0.23* | (0.06-0.94) | 0.30 | (0.05-1.79) | 2.18 | (0.21-22.76) |  |
| *2015* | 1.02 | (0.20-5.33) | 0.21* | (0.05-0.90) | 0.11* | (0.01-0.76) | 1.50 | (0.14-15.84) |  |
| *2016–2020* | 0.73 | (0.14-3.70) | 0.22* | (0.06-0.82) | 0.16* | (0.03-0.94) | 2.33 | (0.23-23.63) |  |
| **Gender (ref. woman)** |  |  |  |  |  |  |  |  |  |
| *Male* | 1.15 | (0.62-2.13) | 1.98 | (0.92-4.26) | 0.58 | (0.23-1.48) | 1.42 | (0.71-2.83) |  |
| **Highest level of education (ref. secondary)** |  |  |  |  |  |  |  |  |  |
| *Primary or lower* | 0.50 | (0.17-1.50) | 2.07 | (0.83-5.19) | 1.30 | (0.33-5.20) | 0.82 | (0.07-10.14) |  |
| *Tertiary* | 0.54 | (0.19-1.52) | 0.44 | (0.12-1.68) | 0.40 | (0.05-3.38) | 2.26 | (0.19-26.81) |  |
| *Don't know/no answer* | 0.80 | (0.41-1.58) | 1.21 | (0.52-2.82) | 2.23 | (0.86-5.80) | 27.56*** | (6.31-120.31) |  |
| **Relationship status (ref. single)** |  |  |  |  |  |  |  |  |  |
| *Partner* | 0.43 | (0.05-3.42) | 0.00 | (0.00-.) | 0.00 | (0.00-.) | 0.00 | (0.00-.) |  |
| *Cohabiting or married* | 0.74 | (0.35-1.57) | 0.62 | (0.25-1.53) | 1.43 | (0.46-4.42) | 1.41 | (0.48-4.18) |  |
| *Divorced, separated or widowed* | 0.59 | (0.17-2.06) | 0.46 | (0.11-1.99) | 0.00 | (0.00-.) | 1.47 | (0.36-6.11) |  |
| *Don't know/no answer* | 1.61 | (0.17-15.18) | 4.62 | (0.63-33.87) | 0.00 | (0.00-.) | 2.68 | (0.49-14.61) |  |
| **Type of place of residence (ref. city)** |  |  |  |  |  |  |  |  |  |
| *Countryside or smaller city* | 1.60 | (0.75-3.43) | 3.40** | (1.38-8.35) | 0.96 | (0.27-3.45) | 0.77 | (0.20-3.05) |  |
| *Big city* | 0.78 | (0.35-1.70) | 2.43* | (1.02-5.80) | 1.29 | (0.47-3.57) | 0.92 | (0.34-2.50) |  |
| *Don't know/no answer* | 1.82 | (0.67-4.97) | 0.59 | (0.07-4.93) | 0.00 | (0.00-.) | 11.43*** | (4.97-26.27) |  |
| **Living with senior (ref. no)** |  |  |  |  |  |  |  |  |  |
| *Yes* | 0.94 | (0.21-4.19) | 1.07 | (0.28-4.19) | 0.88 | (0.10-7.74) | 1.51 | (0.43-5.30) |  |
| *Don't know/no answer* | 1.36 | (0.28-6.62) | 0.00 | (0.00-.) | 1.66 | (0.18-15.40) | 9.33*** | (4.02-21.68) |  |
| Constant | 0.20 | (0.02-2.02) | 0.03* | (0.00-0.50) | 0.18 | (0.01-4.00) | 0.00*** | (0.00-0.02) |  |
| ∗Significant at p < 0.05; ∗∗p < 0.01; ∗∗∗p < 0.001; RRR=relative risk ratio; CIs=confidence intervals; ref.=reference; base category=wants to vaccinate or already vaccinated, n=1,222 | | | | | | | | |  |

Table C added items measuring values and perceptions as well as COVID-19-related factors together, controlling for the sociodemographic factors included in Table 2 (in Table 3 these factors were added one by one in separate models).

| **Table C** Vaccine hesitancy: COVID-19-related factors together | | | | | | | | |  |
| --- | --- | --- | --- | --- | --- | --- | --- | --- | --- |
| **Will get vaccinated:** | **No probably not**  **(n=186)** | | **No definitely not**  **(n=139)** | | **Don't know**  **(n=94)** | | **Don't want to answer**  **(n=192)** | |  |
|  |  |  |  |  |  |  |  |  |  |
|  | RRR | CIs | RRR | CIs | RRR | CIs | RRR | CIs |  |
| **Trust in Swedish authorities (ref. neither big or little)** |  |  |  |  |  |  |  |  |  |
| *Very little* | 0.86 | (0.35-2.07) | 1.41 | (0.64-3.11) | 1.19 | (0.44-3.24) | 1.19 | (0.14-9.99) |  |
| *Quite a bit* | 0.92 | (0.43-2.01) | 0.92 | (0.41-2.06) | 1.17 | (0.43-3.17) | 0.83 | (0.10-7.00) |  |
| *Somewhat high* | 0.92 | (0.47-1.77) | 0.70 | (0.35-1.42) | 0.63 | (0.25-1.59) | 1.10 | (0.17-7.05) |  |
| *Very high* | 0.80 | (0.36-1.75) | 0.75 | (0.31-1.82) | 0.72 | (0.24-2.20) | 2.03 | (0.31-13.18) |  |
| *Don't know* | 1.48 | (0.65-3.40) | 1.13 | (0.46-2.81) | 0.58 | (0.16-2.15) | 2.02 | (0.27-15.00) |  |
| *Don't want to answer* | 1.49 | (0.57-3.88) | 1.21 | (0.39-3.73) | 1.25 | (0.32-4.84) | 9.56* | (1.26-72.79) |  |
| **Importance of vaccination for own health (ref. not important at all)** |  |  |  |  |  |  |  |  |  |
| *Not very important* | 1.21 | (0.43-3.41) | 3.99** | (1.55-10.27) | 0.44 | (0.17-1.12) | 3.52 | (0.55-22.53) |  |
| *Fairly important* | 0.31* | (0.12-0.77) | 0.70 | (0.27-1.78) | 0.05*** | (0.02-0.15) | 0.85 | (0.15-4.75) |  |
| *Very important* | 0.19*** | (0.07-0.48) | 0.15** | (0.05-0.48) | 0.07*** | (0.02-0.18) | 0.21 | (0.03-1.32) |  |
| *Don't know* | 3.99** | (1.62-9.85) | 1.60 | (0.57-4.49) | 0.18** | (0.06-0.55) | 2.32 | (0.40-13.59) |  |
| *Don't want to answer* | 1.19 | (0.37-3.88) | 1.33 | (0.37-4.84) | 0.99 | (0.31-3.19) | 3.32 | (0.56-19.72) |  |
| **Importance of vaccination to protect others (ref. not important at all)** |  |  |  |  |  |  |  |  |  |
| *Not very important* | 17.04* | (1.83-158.49) | 8.63** | (2.25-33.11) | 2.39 | (0.77-7.44) | 1.60 | (0.18-14.20) |  |
| *Fairly important* | 15.28* | (1.88-124.03) | 4.53* | (1.31-15.68) | 1.02 | (0.39-2.66) | 0.81 | (0.15-4.54) |  |
| *Very important* | 5.52 | (0.67-45.28) | 0.81 | (0.22-3.00) | 0.21** | (0.08-0.60) | 0.36 | (0.06-2.00) |  |
| *Don't know* | 39.30*** | (4.67-330.44) | 13.00*** | (3.40-49.69) | 3.12 | (0.97-9.98) | 1.06 | (0.16-6.94) |  |
| *Don't want to answer* | 13.98* | (1.39-141.03) | 9.87** | (1.97-49.36) | 1.02 | (0.24-4.32) | 4.95 | (0.74-33.03) |  |
| **Past COVID-19 infection (ref. no)** |  |  |  |  |  |  |  |  |  |
| *Yes (definitely or maybe)* | 1.21 | (0.76-1.94) | 1.60 | (0.96-2.66) | 1.24 | (0.67-2.30) | 0.34 | (0.10-1.17) |  |
| *Don't know* | 0.91 | (0.52-1.62) | 0.77 | (0.39-1.52) | 0.73 | (0.32-1.69) | 0.39 | (0.10-1.61) |  |
| *Don't want to answer* | 0.60 | (0.25-1.47) | 0.50 | (0.19-1.33) | 0.38 | (0.13-1.17) | 0.94 | (0.30-2.94) |  |
| **Worried about becoming seriously ill in COVID-19 (ref. not at all)** |  |  |  |  |  |  |  |  |  |
| *To some extent* | 1.33 | (0.72-2.44) | 0.66 | (0.36-1.20) | 0.24*** | (0.11-0.54) | 0.71 | (0.17-2.96) |  |
| *Fairly much* | 0.99 | (0.44-2.23) | 0.34* | (0.13-0.87) | 0.85 | (0.31-2.31) | 3.18 | (0.78-12.98) |  |
| *Very much* | 0.55 | (0.20-1.50) | 0.73 | (0.28-1.94) | 0.44 | (0.12-1.53) | 0.46 | (0.06-3.49) |  |
| *Don't know* | 1.46 | (0.61-3.46) | 0.55 | (0.19-1.60) | 1.09 | (0.31-3.81) | 0.94 | (0.12-7.23) |  |
| *Don't want to answer* | 0.66 | (0.13-3.28) | 0.33 | (0.06-1.84) | 0.85 | (0.09-7.97) | 0.32 | (0.03-3.41) |  |
| **Worried about someone close/a relative becoming seriously ill in COVID-19 (ref. not at all)** |  |  |  |  |  |  |  |  |  |
| *To some extent* | 0.88 | (0.39-1.97) | 0.74 | (0.34-1.61) | 0.88 | (0.39-1.99) | 0.36 | (0.07-1.79) |  |
| *Fairly much* | 0.95 | (0.40-2.27) | 0.96 | (0.41-2.27) | 0.44 | (0.15-1.30) | 0.26 | (0.05-1.40) |  |
| *Very much* | 0.79 | (0.32-1.96) | 0.50 | (0.19-1.34) | 0.50 | (0.18-1.38) | 0.78 | (0.16-3.83) |  |
| *Don't know* | 2.03 | (0.71-5.81) | 1.71 | (0.55-5.31) | 0.56 | (0.13-2.46) | 0.84 | (0.09-7.93) |  |
| *Don't want to answer* | 2.24 | (0.43-11.79) | 1.59 | (0.26-9.62) | 0.34 | (0.04-3.30) | 1.50 | (0.14-15.93) |  |
| **Constant** | 0.01*** | (0.00-0.11) | 0.06** | (0.01-0.35) | 1.24 | (0.21-7.37) | 0.02** | (0.00-0.32) |  |
| ∗Significant at p < 0.05; ∗∗p < 0.01; ∗∗∗p < 0.001; RRR=relative risk ratio; CIs=confidence intervals; ref.=reference; base category=wants to vaccinate or already vaccinated; n=2,612; relative risks are displayed net of sociodemographic variables included in Table 2 | | | | | | | | |  |

Tables D–E display results for the alternative dependent variable that measured whether one would recommend others to vaccinate against COVID-19.

Overall, the patterns mirrored the results presented in Tables II and III, with some notable differences. Migrants who had arrived after 2015 were more likely to recommend others to vaccinate. Refusal to answer questions about previous COVID-19 infection was significantly linked to all categories of recommendation hesitation. Those who were worried about themselves or a relative becoming seriously ill had lower opposition towards recommending vaccination.

| **Table D** Would recommend others to vaccinate: sociodemographic factors | | | | | | | | |  |
| --- | --- | --- | --- | --- | --- | --- | --- | --- | --- |
| **Would recommend others to vaccinate:** | **No probably not**  **(n=110)** | | **No definitely not**  **(n=147)** | | **Don't know**  **(n=208)** | | **Don't want to answer**  **(n=283)** | |  |
|  |  |  |  |  |  |  |  |  |  |
|  | RRR | CIs | RRR | CIs | RRR | CIs | RRR | CIs |  |
| **Age group (ref. 35–44)** |  |  |  |  |  |  |  |  |  |
| *Under 25* | 1.78 | (0.86-3.69) | 1.45 | (0.76-2.74) | 1.26 | (0.74-2.14) | 0.76 | (0.40-1.42) |  |
| *25–34* | 1.21 | (0.70-2.09) | 0.90 | (0.57-1.43) | 1.16 | (0.80-1.68) | 0.97 | (0.63-1.49) |  |
| *45–54* | 0.75 | (0.40-1.41) | 0.62 | (0.37-1.05) | 0.51** | (0.31-0.84) | 0.79 | (0.47-1.34) |  |
| *55+* | 0.86 | (0.42-1.75) | 0.43* | (0.22-0.84) | 0.26*** | (0.13-0.54) | 0.60 | (0.32-1.13) |  |
| **Country of birth (ref. Western Europe, North America, Australia or New Zealand)** |  |  |  |  |  |  |  |  |  |
| *MENA* | 0.61 | (0.22-1.68) | 0.94 | (0.36-2.51) | 1.83 | (0.63-5.27) | 0.42 | (0.16-1.07) |  |
| *SSA* | 0.91 | (0.32-2.59) | 0.79 | (0.28-2.20) | 1.18 | (0.40-3.50) | 0.50 | (0.19-1.28) |  |
| *Eastern Europe* | 2.50 | (0.77-8.20) | 3.92* | (1.32-11.61) | 2.61 | (0.74-9.17) | 1.01 | (0.30-3.34) |  |
| *South America* | 2.60 | (0.87-7.78) | 1.25 | (0.38-4.11) | 1.06 | (0.27-4.19) | 0.24 | (0.04-1.31) |  |
| *Asia* | 0.50 | (0.14-1.84) | 0.91 | (0.30-2.77) | 0.71 | (0.20-2.58) | 0.48 | (0.14-1.62) |  |
| **Year of arrival (ref. 2007 or earlier)** |  |  |  |  |  |  |  |  |  |
| *2008–2014* | 0.94 | (0.49-1.80) | 0.63 | (0.35-1.11) | 0.72 | (0.42-1.24) | 0.92 | (0.44-1.93) |  |
| *2015* | 0.59 | (0.28-1.25) | 0.59 | (0.32-1.09) | 0.74 | (0.43-1.29) | 1.14 | (0.54-2.38) |  |
| *2016–2020* | 0.44* | (0.22-0.86) | 0.50* | (0.29-0.87) | 0.64 | (0.38-1.07) | 1.42 | (0.71-2.81) |  |
| **Gender (ref. woman)** |  |  |  |  |  |  |  |  |  |
| *Male* | 1.22 | (0.82-1.82) | 0.70* | (0.49-0.99) | 1.04 | (0.77-1.40) | 1.10 | (0.79-1.54) |  |
| **Highest level of education (ref. secondary)** |  |  |  |  |  |  |  |  |  |
| *Primary or lower* | 1.69 | (0.86-3.32) | 1.49 | (0.80-2.78) | 1.34 | (0.80-2.25) | 1.01 | (0.38-2.67) |  |
| *Tertiary* | 1.17 | (0.69-1.99) | 0.94 | (0.59-1.49) | 0.84 | (0.54-1.29) | 1.27 | (0.59-2.71) |  |
| *Don't know/no answer* | 1.45 | (0.84-2.50) | 1.61* | (1.03-2.54) | 1.77** | (1.24-2.54) | 15.30*** | (9.16-25.56) |  |
| **Relationship status (ref. single)** |  |  |  |  |  |  |  |  |  |
| *Partner* | 0.27 | (0.06-1.18) | 0.83 | (0.33-2.10) | 0.76 | (0.34-1.69) | 0.55 | (0.16-1.87) |  |
| *Cohabiting or married* | 0.82 | (0.48-1.38) | 0.87 | (0.53-1.44) | 0.72 | (0.48-1.06) | 0.95 | (0.59-1.52) |  |
| *Divorced, separated or widowed* | 0.66 | (0.29-1.51) | 1.71 | (0.91-3.23) | 0.98 | (0.56-1.72) | 0.70 | (0.36-1.39) |  |
| *Don't know/no answer* | 0.00 | (0.00-.) | 2.47 | (0.65-9.39) | 0.84 | (0.18-3.90) | 2.02 | (0.71-5.72) |  |
| **Type of place of residence (ref. city)** |  |  |  |  |  |  |  |  |  |
| *Countryside or smaller city* | 1.02 | (0.55-1.89) | 1.15 | (0.67-1.99) | 1.16 | (0.74-1.81) | 1.17 | (0.67-2.06) |  |
| *Big city* | 0.99 | (0.63-1.55) | 1.17 | (0.80-1.73) | 0.95 | (0.67-1.34) | 1.29 | (0.85-1.95) |  |
| *Don't know/no answer* | 0.95 | (0.36-2.50) | 0.73 | (0.28-1.92) | 1.60 | (0.90-2.83) | 7.54*** | (4.73-12.03) |  |
| **Living with senior (ref. no)** |  |  |  |  |  |  |  |  |  |
| *Yes* | 0.78 | (0.33-1.88) | 1.48 | (0.77-2.85) | 0.93 | (0.47-1.85) | 1.31 | (0.66-2.60) |  |
| *Don't know/no answer* | 1.09 | (0.24-4.87) | 0.42 | (0.05-3.22) | 0.38 | (0.09-1.66) | 5.33*** | (2.88-9.87) |  |
| **Constant** | 0.09*** | (0.03-0.30) | 0.13*** | (0.04-0.43) | 0.12*** | (0.04-0.39) | 0.03*** | (0.01-0.11) |  |
| ∗Significant at p < 0.05; ∗∗p < 0.01; ∗∗∗p < 0.001; RRR=relative risk ratio; CIs=confidence intervals; ref.=reference; base category=wants to vaccinate or already vaccinated; n=2,612 | | | | | | | | |  |

| **Table E** Would recommend others to vaccinate: COVID-19-related factors | | | | | | | | |  |
| --- | --- | --- | --- | --- | --- | --- | --- | --- | --- |
| **Would recommend others to vaccinate:** | **No probably not**  **(n=110)** | | **No definitely not**  **(n=147)** | | **Don't know**  **(n=208)** | | **Don't want to answer**  **(n=283)** | |  |
|  |  |  |  |  |  |  |  |  |  |
|  | RRR | CIs | RRR | CIs | RRR | CIs | RRR | CIs |  |
| **Trust in Swedish authorities (ref. neither big or little)** |  |  |  |  |  |  |  |  |  |
| *Very little* | 0.39* | (0.16-0.94) | 1.90 | (1.00-3.62) | 0.35* | (0.15-0.78) | 2.61 | (0.73-9.30) |  |
| *Quite a bit* | 0.70 | (0.35-1.41) | 1.08 | (0.54-2.16) | 0.94 | (0.54-1.65) | 2.24 | (0.63-8.01) |  |
| *Somewhat high* | 0.49* | (0.27-0.88) | 0.84 | (0.46-1.52) | 0.51* | (0.30-0.86) | 1.31 | (0.40-4.29) |  |
| *Very high* | 0.18*** | (0.07-0.44) | 0.39* | (0.18-0.87) | 0.54* | (0.30-0.95) | 0.39 | (0.07-2.16) |  |
| *Don't know* | 0.98 | (0.43-2.21) | 1.48 | (0.64-3.41) | 1.86* | (1.04-3.33) | 1.69 | (0.39-7.39) |  |
| *Don't want to answer* | 0.89 | (0.33-2.36) | 1.60 | (0.68-3.80) | 0.76 | (0.38-1.51) | 18.88*** | (5.49-64.90) |  |
| Constant | 0.09*** | (0.03-0.29) | 0.14*** | (0.05-0.37) | 0.11*** | (0.04-0.31) | 0.01*** | (0.00-0.04) |  |
| **Importance of vaccination for own health (ref. not important at all)** |  |  |  |  |  |  |  |  |  |
| *Not very important* | 3.30** | (1.42-7.66) | 2.04* | (1.02-4.05) | 1.82 | (0.82-4.06) | 1.33 | (0.28-6.26) |  |
| *Fairly important* | 0.82 | (0.38-1.77) | 0.29*** | (0.15-0.56) | 0.81 | (0.42-1.56) | 0.62 | (0.20-1.92) |  |
| *Very important* | 0.14*** | (0.06-0.31) | 0.10*** | (0.05-0.19) | 0.19*** | (0.10-0.38) | 0.11*** | (0.03-0.39) |  |
| *Don't know* | 1.68 | (0.67-4.20) | 1.74 | (0.88-3.46) | 6.20*** | (3.20-12.02) | 3.15* | (1.02-9.75) |  |
| *Don't want to answer* | 3.26* | (1.02-10.40) | 2.49 | (0.99-6.25) | 2.64* | (1.01-6.89) | 129.92*** | (42.78-394.59) |  |
| Constant | 0.07*** | (0.02-0.27) | 0.36 | (0.12-1.06) | 0.10*** | (0.03-0.33) | 0.01*** | (0.00-0.07) |  |
| **Importance of vaccination to protect others (ref. not important at all)** |  |  |  |  |  |  |  |  |  |
| *Not very important* | 2.99* | (1.13-7.92) | 5.09*** | (2.14-12.12) | 7.84** | (2.21-27.82) | 0.86 | (0.08-8.70) |  |
| *Fairly important* | 0.88 | (0.41-1.88) | 0.77 | (0.38-1.58) | 2.68 | (0.92-7.81) | 0.43 | (0.12-1.55) |  |
| *Very important* | 0.09*** | (0.04-0.20) | 0.10*** | (0.05-0.22) | 0.68 | (0.24-1.97) | 0.14** | (0.04-0.49) |  |
| *Don't know* | 1.66 | (0.64-4.32) | 3.75** | (1.68-8.37) | 23.62*** | (7.87-70.87) | 3.26 | (0.91-11.64) |  |
| *Don't want to answer* | 1.75 | (0.40-7.67) | 3.42* | (1.10-10.66) | 5.65* | (1.28-24.89) | 200.88*** | (54.25-743.79) |  |
| Constant | 0.12** | (0.03-0.48) | 0.30* | (0.10-0.94) | 0.05*** | (0.01-0.19) | 0.01*** | (0.00-0.07) |  |
| **Past COVID-19 infection (ref. no)** |  |  |  |  |  |  |  |  |  |
| *Yes (definitely or maybe)* | 1.45 | (0.91-2.33) | 1.24 | (0.80-1.92) | 1.09 | (0.76-1.58) | 1.34 | (0.70-2.57) |  |
| *Don't know* | 1.00 | (0.52-1.91) | 1.45 | (0.88-2.38) | 1.25 | (0.80-1.94) | 0.83 | (0.31-2.20) |  |
| *Don't want to answer* | 2.37* | (1.06-5.29) | 2.97** | (1.52-5.80) | 2.38** | (1.23-4.58) | 84.11*** | (47.64-148.47) |  |
| Constant | 0.04*** | (0.01-0.12) | 0.09*** | (0.04-0.23) | 0.06*** | (0.02-0.17) | 0.00*** | (0.00-0.01) |  |
| **Worried about becoming seriously ill in COVID-19 (ref. not at all)** |  |  |  |  |  |  |  |  |  |
| *To some extent* | 0.41** | (0.24-0.70) | 0.47** | (0.30-0.74) | 0.58* | (0.38-0.88) | 0.63 | (0.29-1.40) |  |
| *Fairly much* | 0.41** | (0.21-0.81) | 0.30*** | (0.16-0.58) | 0.46** | (0.26-0.80) | 0.60 | (0.22-1.65) |  |
| *Very much* | 0.48* | (0.24-0.96) | 0.31*** | (0.16-0.62) | 0.49* | (0.27-0.90) | 0.53 | (0.17-1.70) |  |
| *Don't know* | 0.84 | (0.35-2.01) | 1.23 | (0.63-2.40) | 2.34** | (1.38-3.97) | 1.59 | (0.55-4.59) |  |
| *Don't want to answer* | 1.05 | (0.45-2.45) | 0.60 | (0.29-1.23) | 1.45 | (0.81-2.59) | 17.95*** | (7.97-40.43) |  |
| Constant | 0.08*** | (0.02-0.25) | 0.23** | (0.09-0.57) | 0.11*** | (0.04-0.29) | 0.02*** | (0.00-0.07) |  |
| **Worried about someone close/a relative becoming seriously ill in COVID-19 (ref. not at all)** |  |  |  |  |  |  |  |  |  |
| *To some extent* | 0.64 | (0.32-1.28) | 0.39*** | (0.23-0.67) | 0.54* | (0.31-0.95) | 0.47 | (0.16-1.33) |  |
| *Fairly much* | 0.46* | (0.22-0.97) | 0.28*** | (0.16-0.51) | 0.47* | (0.26-0.86) | 0.44 | (0.15-1.28) |  |
| *Very much* | 0.35** | (0.17-0.75) | 0.16*** | (0.08-0.29) | 0.55* | (0.31-0.97) | 0.48 | (0.17-1.37) |  |
| *Don't know* | 1.74 | (0.71-4.25) | 0.93 | (0.43-2.00) | 2.90** | (1.51-5.54) | 0.87 | (0.20-3.72) |  |
| *Don't want to answer* | 1.17 | (0.47-2.96) | 0.36** | (0.17-0.76) | 1.25 | (0.64-2.43) | 15.62*** | (5.73-42.55) |  |
| Constant | 0.08*** | (0.02-0.29) | 0.36* | (0.14-0.96) | 0.12*** | (0.04-0.35) | 0.03*** | (0.01-0.11) |  |
| ∗Significant at p < 0.05; ∗∗p < 0.01; ∗∗∗p < 0.001; RRR=relative risk ratio; CIs=confidence intervals; ref.=reference; base category=wants to vaccinate or already vaccinated; n=2,612; relative risks are displayed net of sociodemographic variables included in Table C | | | | | | | | |  |
